# Co-Translational Folding of Multi-Domain Proteins

**DOI:** 10.3389/fmolb.2022.869027

**Published:** 2022-04-20

**Authors:** Nandakumar Rajasekaran, Christian M. Kaiser

**Affiliations:** ^1^ CMDB Graduate Program, Johns Hopkins University, Baltimore, MD, United States; ^2^ Department of Biology, Johns Hopkins University, Baltimore, MD, United States; ^3^ Department of Biophysics, Johns Hopkins University, Baltimore, MD, United States

**Keywords:** co-translational folding, multi-domain proteins, single-molecule methods, optical tweezers, inter-domain interactions, protein misfolding, ribosome

## Abstract

The majority of proteins in nature are composed of multiple domains connected in a single polypeptide. How these long sequences fold into functional structures without forming toxic misfolds or aggregates is poorly understood. Their folding is inextricably linked to protein synthesis and interactions with cellular machinery, making mechanistic studies challenging. Recent progress has revealed critical features of multi-domain protein folding in isolation and in the context of translation by the ribosome. In this review, we discuss challenges and progress in understanding multi-domain protein folding, and highlight how molecular interactions shape folding and misfolding pathways. With the development of new approaches and model systems, the stage is now set for mechanistically exploring the folding of large multi-domain proteins.

## Multi-domain Proteins

Proteins exhibit an astonishing functional diversity, playing essential roles in all cellular processes. Strikingly, this remarkable diversity is achieved with a surprisingly small number of building blocks. So far, approximately 1,500 unique folds have been identified in all known structures (Andreeva et al., 2020). The vast diversity observed in extant proteomes is achieved by combining domains (Doolittle, 1995) into a single polypeptide. The majority of proteins in all organisms are composed of multiple constituent domains, with some eukaryotic proteomes having their proportion of multi-domain proteins reach as much as four fifths (Han et al., 2007). Stringing together multiple domains into a single polypeptide chain therefore appears to be a successful evolutionary strategy to create proteins with novel functions and might also be attractive for designing new functions for synthetic applications (Poust et al., 2014; Kim et al., 2015a).

**FIGURE 1:**
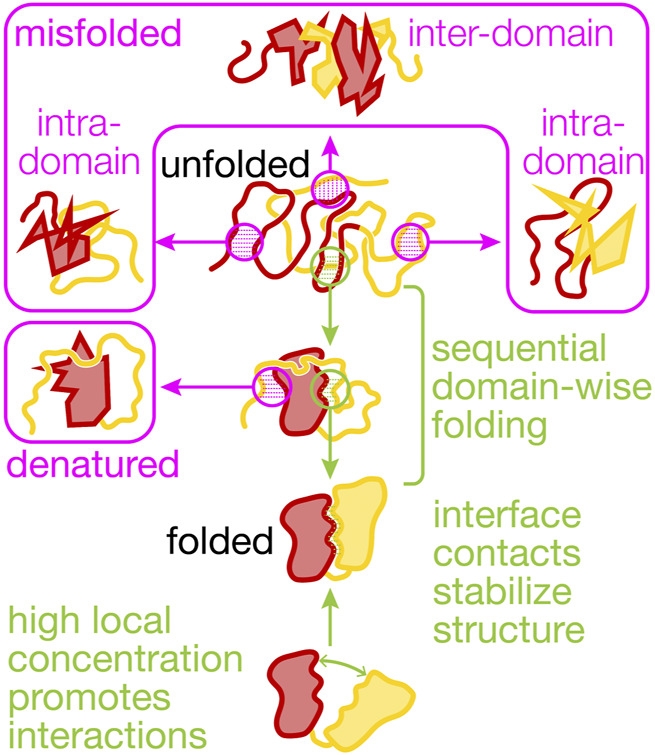
Productive and non-productive interactions between domains in a protein. The cartoons visualize non-productive interactions (magenta) and productive interactions (green) within and between two domains (N-terminal, red, and C-terminal, yellow) that shape multi-domain (mis)folding and dynamics. Co-translational folding abets sequential domain-wise folding, reducing inter-domain misfolding. Interactions with the ribosome and molecular chaperones further reduce misfolding and the destabilizing interactions between the unfolded yellow and the folded red domain that results in denaturation. Non-native states are prone to irreversible aggregation (not shown here). Interface contacts between the folded domains can be mutually stabilizing and are promoted by high local concentrations that result from covalently linking the domains in a single polypeptide chain.

Interactions between domains in a large protein (Figure 1) are conceptually similar to the protein-protein interactions that, with their finely tuned specificities and affinities, are a main driver of biological complexity at the molecular level (Huttlin et al., 2021). This complexity makes detailed biophysical studies of multi-domain protein folding and dynamics challenging. Among the best-characterized models for interactions between domains that are connected by a linker are the eye lens crystallins, for which domain coupling has been elucidated in detail [reviewed in (Jaenicke, 1999)]. Recently, it was also shown that the folding of gamma-B crystallin is directed by early co-translational events (Buhr et al., 2016).

Connecting domains covalently into a single polypeptide results in very high local concentrations. For example, two domains connected by a flexible glycine-serine linker of 20 amino acids have an effective concentration, defined as the concentration required to achieve the same encounter rate between two untethered domains, of 4 mM (Sørensen and Kjaergaard, 2019). The identity of the intervening linker sequence affects the local concentration effect (Sørensen and Kjaergaard, 2019; Kjaergaard, 2022), providing a way of potentially modulating these interactions, as has also been shown for the crystallins (Jaenicke, 1999). Combining two domains together thus increases their likelihood of interacting between two domains (Sivaramakrishnan and Spudich, 2011), which may tune the rate of catalyzed reactions (Dyla and Kjaergaard, 2020). The enhanced encounter rate may be crucial for stabilizing protein domains (Robinson and Sauer, 1998; Jäger and Plückthun, 1999) and to promote proximity between functional units.

## Experimental Approaches

One potential evolutionary limitation of stringing together domains into proteins with novel functions is the requirement that the resulting multi-domain protein must be able to fold into a functional structure (Han et al., 2007). Indeed, misfolding between unfolded domains has been demonstrated to significantly challenge the refolding of multi-domain proteins *in vitro* (Fernandez et al., 1999; Borgia et al., 2011; Jahn et al., 2016; Liu et al., 2017), making experimental studies challenging. Misfolding, aggregation and the formation of partially folded intermediates result in complex measured signals that are difficult to resolve with the traditional methods that have been highly successful for studying small proteins or individual domains (Bartlett and Radford, 2009). In addition, averaging over ensembles of stochastically folding molecules makes it challenging to resolve individual steps in the multi-step folding pathways of large proteins. Single-molecule approaches are a particularly promising avenue toward resolving the folding of larger multi-domain proteins.

Following the folding of individual molecules circumvents the problems of aggregation and ensemble averaging. Single-molecule fluorescence methods have allowed direct visualization of different conformations of multi-domain proteins at equilibrium (Huang et al., 2009) and measurement of global refolding kinetics (Borgia et al., 2011). Force spectroscopy approaches, with their ability to selectively perturb the stability of specific domains while leaving the rest of the protein folded, are ideally suited to dissect multi-domain protein folding. Single-molecule manipulation with optical tweezers permits the detailed characterization of energetics and kinetics (Bustamante et al., 2020). Importantly, it also provides a means of observing the folding of a protein of interest against the complex backdrop of the molecular machinery that is crucial for efficient folding of multi-domain proteins.

## Co-Translational Folding

The mode of cellular protein synthesis is a key factor for the folding of multi-domain proteins and, therefore, their evolution. Phillips noted five decades ago that “it seems useful to note that protein molecules are synthesized from their terminal amino ends [...] and to suggest that the folding process may begin during synthesis.” (Phillips, 1967). At this time, co-translational folding (and assembly into a functional tetramer) had already been suggested by detecting enzymatic activity of ribosome-bound *β*-galactosidase (Zipser and Perrin, 1963). Subsequently, co-translational folding was demonstrated for a number of proteins, including immunoglobulin domains (Bergman and Kuehl, 1979) and firefly luciferase (Frydman et al., 1994) (see Fedorov and Baldwin, 1997 for a review). The slow addition of amino acids by the ribosome during translation of the genetic information gives the nascent protein ample time to populate stably folded intermediates. Timely structuring of emerging nascent chain segments reduces the accumulation of unfolded domains, thus enabling temporal sequestration of the folding of individual domains and counteracting misfolding. Recently, interactions with the ribosome and with molecular chaperones were shown to be crucial for efficiently producing functional multi-domain proteins (Deuerling et al., 2019; Kramer et al., 2019; Liutkute et al., 2020a; Cassaignau et al., 2020; Maciuba et al., 2021). Experimentally studying the process requires overcoming significant challenges (see below), and the number of folding studies analyzing multi-domain proteins is still small compared to those investigating single-domain proteins (Braselmann et al., 2013). Here, we discuss some of the recent progress that has been made toward defining principles of multi-domain protein folding both in the context of translation by the ribosome and in isolation.

The ribosome is itself a large complex of several megadaltons made of RNA and protein. Most spectroscopic techniques cannot discern the signal from the folding nascent chain against the backdrop of the ribosome. New NMR methodologies employing selective isotope labeling of nascent proteins and novel pulse sequences (Cassaignau et al., 2016) have provided high-resolution structural information of nascent chain folding and dynamics (Waudby et al., 2013). However, they require high sample concentrations and are thus far limited to relatively short nascent proteins. Fluorescence methods such as FRET have allowed direct visualization of different conformations of the nascent chain populated during translation and resolve folding kinetics. Combined FRET and PET measurements were used to show that nascent chains can form secondary structural elements even inside the exit tunnel (Holtkamp et al., 2015; Liutkute et al., 2020b). Force spectroscopy techniques also allow detection of nascent chain folding on the ribosome. In addition, application of defined, variable mechanical force can be used to selectively destabilize nascent protein structure, keeping interacting partners such as the ribosome intact, in contrast to globally acting chemical or thermal denaturation. Force spectroscopy can thus be used to drive non-equilibrium transitions and to study the folding dynamics of nascent chains in the presence of biologically relevant interaction partners.

Because of the substantial challenges associated with implementing, conducting, and analyzing experiments of co-translational folding, initial studies were carried out using single-domain proteins with artificial C-terminal extensions that allowed their full extrusion from the ribosome (Kaiser et al., 2011; Knight et al., 2013; Samelson et al., 2016; Guinn et al., 2018; Waudby et al., 2018; Liu et al., 2019a). While these experiments did not capture the complexity of naturally occurring large proteins, they yielded several key insights about the interactions of emerging domains with the ribosome that are directly relevant to multi-domain protein folding. In particular, single-molecule optical tweezers experiments revealed that interactions with the ribosome generally decelerate and destabilize nascent chain folding, presumably due to electrostatic interactions with the surface of the ribosome that decay as the nascent chain is elongated (Kaiser et al., 2011). The distance-dependent deceleration of domain folding is likely the result of competition between mutually exclusive interactions of the nascent chain with itself (resulting in folding) and with the ribosome surface (preventing folding). These interactions may help to stabilize a folding-competent conformation of the nascent protein during translation before a foldable unit has been synthesized and extruded from the ribosome.

The ability to precisely monitor changes in molecular length with optical tweezers enables real time observation of nascent chain elongation and folding. The addition of individual amino acids is difficult to resolve because the change in molecular extension per amino acid added is very small (less than ∼2 Å at forces below 10 pN) relative to the noise of an optical tweezers measurement. However, overall chain elongation is apparent as an increase in length of the tethered molecule. Gradual hydrophobic collapse and partial folding was observed during synthesis of a small single-domain protein in the optical tweezers (Wruck et al., 2017). Studies of the calcium-binding protein calerythrin showed that the protein populates a misfolded state on stalled ribosomes in a few seconds. When the elongation and folding of the nascent chain is monitored in real time, the protein does not populate the misfolded state until tens of seconds have elapsed (Alexander et al., 2019). This study illustrates that the nascent chain does not necessarily equilibrate rapidly, and that non-equilibrium effects may suppress formation of misfolded states.

## Misfolding Interactions

### Inter-Domain Misfolding

Interactions between residues in separate unfolded domains can lead to misfolding (Figure 1). A tandem-repeat protein constructed from the titin I27 domain exhibits inter-domain misfolding after mechanical unfolding (Fernandez et al., 1999). In an artificial construct with repeats of maltose-binding protein, stable misfolds involving multiple domains are observed to form that require high forces to unfold (Mashaghi et al., 2016). Repeats sharing a high sequence similarity are particularly prone to forming very stable misfolded structures, having a lifetime of up to several days (Borgia et al., 2011). At least for the small Ig-like beta-sandwich domains like I27 that have been studied in detail, these structures can form when strands from different domains are swapped between domains. Contiguous domains having lower similarity, however, are less likely to form misfolded structures.

However, inter-domain misfolding is not limited to the special case of repeat proteins. Even relatively small proteins, such as the calcium-binding protein calmodulin and NCS-1, populate non-native structures that compete with productive folding (Stigler et al., 2011; Heidarsson et al., 2014). The three domains of 709-residue long Hsp90 form several inter-domain misfolded structures (Jahn et al., 2016). The structurally complex luciferase protein also populates misfolded states (Scholl et al., 2014). Elongation factor G, a 704-residue long protein consisting of five non-homologous domains forms many stable non-native structures upon refolding (Liu et al., 2017). At least some of these structures involve residues from multiple domains that misfold into mechanically labile, molten globule-like structures. Even in truncated constructs containing only the first two domains, inter-domain misfolding is observed (Liu et al., 2019a). Only a small number of mechanistic studies are available to date. However, inter-domain misfolding into loosely structured misfolded states (for natural multi-domain proteins) appears to be a general phenomenon (Zheng et al., 2013; Tian and Best, 2016).

**FIGURE 2:**
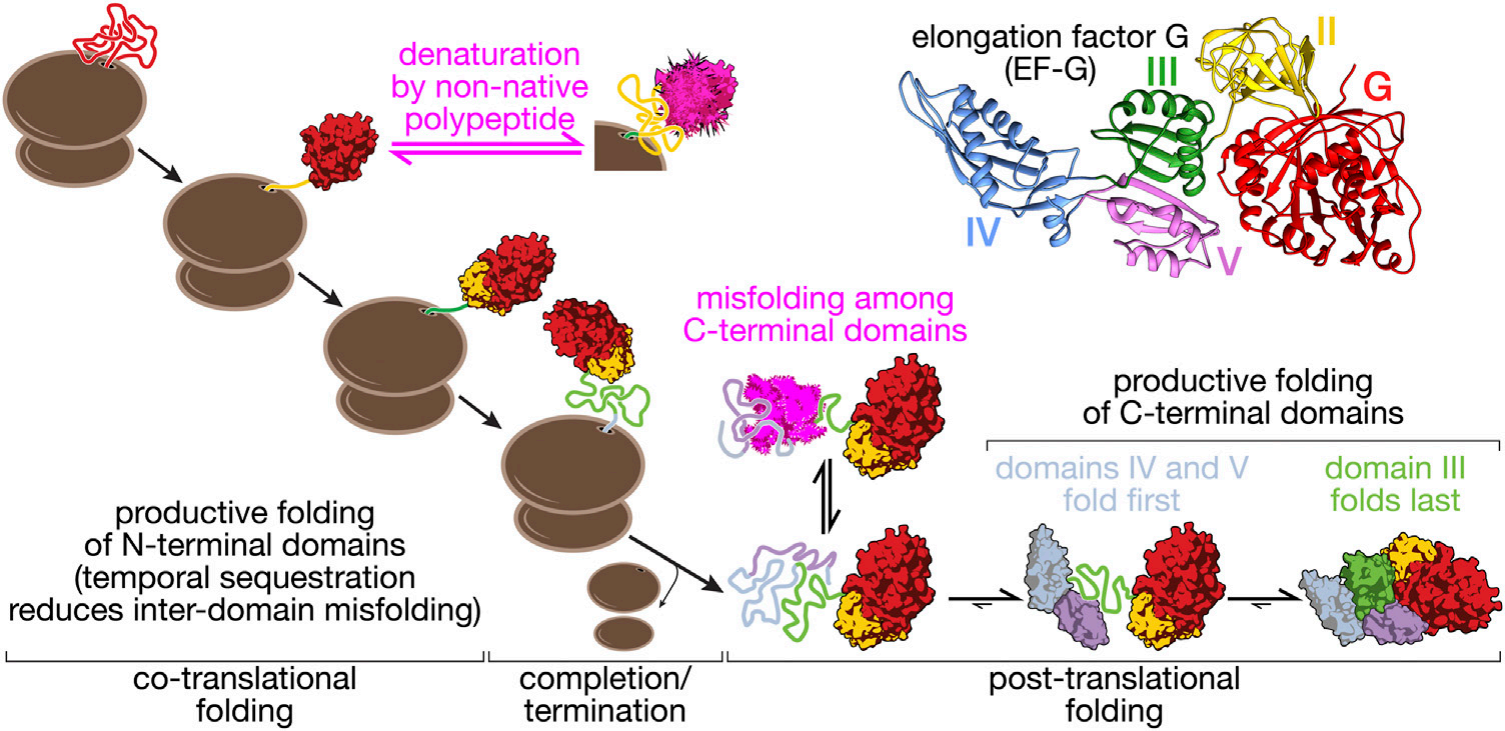
Folding pathway of elongation factor G (EF-G). EF-G is a five-domain protein (structure, top right: pdb 4v9p) that illustrates some of the core aspects of multi-domain protein folding. The N-terminal domains (G, II) fold co-translationally, with G-domain folding being a prerequisite for domain II folding. Productive folding competes with denaturation of the G-domain through interactions with unfolded domain II. This complication of co-translational folding is mitigated by a nascent chain-binding chaperone (Liu et al., 2019a). Because domain III requires stabilizing contacts with domains IV and V, co-translational folding is disrupted. Post-translational folding of the C-terminal domains (III–V) results in the accumulation of unfolded polypeptide during EF-G synthesis that has a high propensity to form off-pathway misfolded states (Liu et al., 2019b). EF-G thus provides an example of how overall folding pathways are shaped by domain interactions in large proteins.

The results outlined in the preceding paragraphs were obtained with isolated proteins and suggest a general propensity of large proteins to populate non-native structures. Nascent chains appear to be at least partially protected from this type of misfolding. For EF-G, misfolding interactions are greatly reduced by the ribosome, promoting efficient sequential folding of the two N-terminal domains G and II (Liu et al., 2019a) (Figure 2). The importance of productive co-translational folding is illustrated by the fact that cells have evolved not only to contain an armory of molecular chaperones that facilitate folding (Balchin et al., 2016; Koubek et al., 2021), but also mechanisms for detecting misfolded states that result in nascent chain degradation (Brandman et al., 2012; Wang et al., 2013) or termination of translation (Zhao et al., 2021). The temporal sequestration of folding during synthesis allows domains to fold into stable structures and prevents non-native interactions. Translation elongation has been suggested to be slow at domain boundaries (Komar, 2009), which would amplify this effect. More generally, translation elongation is likely tuned to allow productive folding (Komar, 2016), as indicated by pronounced effects that synonymous codon changes can have on protein structure, stability and, potentially, function (Kimchi-Sarfaty et al., 2007; Komar, 2009; Zhou et al., 2013; Sander et al., 2014; Kim et al., 2015b; Buhr et al., 2016; Walsh et al., 2020) (see (Liu et al., 2021) for a recent review).

### Intra-Domain Misfolding

In addition to inter-domain misfolding interactions, constituent domains in complex proteins can suffer from internal misfolding (Figure 1). Modeling suggests how tuning of elongation rates by natural selection can help co-translational folding by circumventing kinetic traps (Bitran et al., 2020). Experimentally, individual domains of Hsp90 form misfolded states, in addition to forming inter-domain misfolds (Jahn et al., 2016). The glucocorticoid receptor ligand binding domain populates an ensemble of misfolded states which compete with productive folding (Suren et al., 2018). Application of mechanical force to the molecule reduces misfolding, presumably because mechanically stretching the protein disfavors intra-chain interactions that result in misfolding. Interestingly, folding of nascent proteins close to the ribosomal exit tunnel generates mechanical force (Goldman et al., 2015; Maciuba et al., 2021). The tension generated in the process could similarly destabilize misfolded states and promote the formation of native structures, although this possibility remains to be experimentally tested.

The G-domain of EF- G populates unstable molten-globule like states and intermediate structures upon refolding. When the protein is synthesized, however, the nascent chain remains unfolded until the entire domain is extruded from the ribosome, avoiding the formation of non-native structures (Chen et al., 2020). The resulting directional folding in the C- to N-terminal direction may be prevalent for domains that are prone to misfolding, as illustrated by several examples. The N-terminal domain of calerythrin folds into stable structures in isolation but fails to fold on the ribosome until the entire protein is synthesized (Alexander et al., 2019). The nucleotide binding domain of Hsp70 folds through a folding nucleus involving the C-terminal lobes (Bauer et al., 2018) and may thus remain unstructured until the entire domain is fully extruded from the ribosome during translation. Other domains fold in the opposite direction, such as the nucleotide binding domain of CFTR (Khushoo et al., 2011; Kim et al., 2015b).

Intermediates are commonly thought to be populated on the way to native state, even for relatively small domains (Brockwell and Radford, 2007). It is intuitive to assume that the sequential addition of amino acids to the growing polypeptide will result in gradual compaction and formation of hierarchical structures but, as the examples discussed above demonstrate, the directionality of folding can run counter to that of synthesis. It is interesting to note that folding of the nucleotide binding domains appears to nucleate around the ligand binding site for both CFTR and Hsp70. However, the nucleation sites are located at the N-terminus and C-terminus of the domains, respectively, which might explain why they exhibit differences in their co-translational folding properties. Regardless of the underlying causes, the examples discussed above (EF-G, Hsp70, and calerythrin) strongly suggest that caution is in order when relating folding pathways determined with full-length domains *in vitro* to the co-translational pathways that are likely relevant *in vivo*.

## Stabilizing Interactions

Protein segments rich in low complexity sequences remain intrinsically disordered in solution (van der Lee et al., 2014), unlike globular proteins that are thought to mostly adopt more or less well-defined structures. IDPs have defined sequence features distinct from non-IDPs. However, whether a member of either class is structured or unstructured can depend on context and interactions. Domains in larger globular proteins may not be stable by themselves, and IDPs may adopt defined structures. For instance, binding of intrinsically disordered proteins to their ligands or interacting partners promotes structure formation (Wright and Dyson, 2009). The phosphorylated kinase inducible activation domain of the transcription factor CREB is intrinsically disordered in solution and forms alpha helical structure upon binding to the KIX domain (Sugase et al., 2007). The nuclear coactivator-binding domain of CBP adopts stable structures by interactions with ACTR domain (Zosel et al., 2018). The close association through a stabilizing interface favors the folded conformations of the disordered domains.

The domain interfaces in a multi-domain protein provide opportunities for favorable interactions that stabilize unstable structures (Figure 1). Such stabilizing effects can even promote structure formation in otherwise unstructured domains. For instance, the isolated C-terminal domain of phosphoglycerate kinase remains disordered in solution but is stably folded in the presence of the N-terminal domain (Young et al., 2007). In EF-G, domain II appears to rely on contacts with the N-terminal G-domain for stability (Liu et al., 2019a). In another example, also from EF-G, the central domain III does not appear to be stably folded in isolation, lacking stable tertiary structure (Liu et al., 2019b). Domain III forms extensive interface with its neighboring domains, IV and V. These interfacial contacts stabilize the folded state relative to the unfolded state and the domain is fully folded in the presence of folded domains IV and V (Liu et al., 2019b) (Figure 2).

This stabilization by interaction may be analogous to the ‘binding and folding’ model observed for several disordered proteins. For example, ACTR is largely unstructured in solution and binding to its partner, NCBD, enables it to adopt tertiary structure (Demarest et al., 2002; Marino et al., 2018; Zosel et al., 2018). Another example is the PUMA protein that adopts a helical structure only when binding to MCL-1 (Rogers et al., 2014). A folded domain may also provide an interaction surface that accelerates the folding of a yet unstructured neighbor. The spectrin repeat R17 folds faster when it is stabilized by the upstream domain R16 whereas it folds more slowly in isolation (Batey et al., 2006). Conversely, the unfolding rate of R17 is slower by two orders of magnitude in the presence of stabilizing interactions with R16. The scaffolding effect provided by domain interfaces and modulation of folding rates may aid in productive structure formation. Interestingly, some protein complexes appear to require assembly in a sequential order (Marsh et al., 2013). It seems possible that the folding order prescribed by the directionality of protein synthesis may enforce a similarly ordered formation of domain interfaces in multi-domain proteins that ultimately serves efficient folding.

Not all domain-domain interfaces have stabilizing effects. An extensive interface of domain III in EF-G with its N-terminal neighbors (domains G and II) does not contribute measurably to stability (Liu et al., 2019b). The implications of this complex energetic dependency to the biogenesis of the protein are two-fold. First, domain III will be synthesized before the emergence of domains IV-V, shifting the folding from co- to post- translational regime (Figure 2). Second, the presence of a disordered sequence during synthesis increases the probability of misfolding of emerging nascent chain segments, thus requiring the aid of chaperones. The fusion of multiple domains in a multi-domain protein may stabilize structures that are unstable in isolation. In some cases, like that of domain III of EFG, the energetic dependencies may have evolved to meet functional needs for flexibility. The complex energetic interactions between different domains may pose problems to co-translational folding.

## Denaturation and Destabilizing Interactions

Nascent proteins can begin to acquire secondary and (limited) tertiary structure even before their emergence from the ribosome exit tunnel (Nilsson et al., 2015; Marino et al., 2016; Tian et al., 2018; Agirrezabala et al., 2022). These early structures might be important stepping stones toward the native state. Large structures cannot be accommodated inside the ribosome exit tunnel. Because protein folding is typically cooperative, these structures are fully formed only when an entire domain has emerged from the ribosome. Non-native nascent chain segments therefore are inevitably present during protein synthesis. It has been shown that they can destabilize already folded domains (Figure 1). Unfolded domain II of EF-G destabilizes the folded G-domain, ultimately resulting in its denaturation (Liu et al., 2019a) (Figure 2). Sequestering the unfolded segments near the ribosomal surface may help to reduce denaturing interactions. However, in the case of nascent EF-G, the ribosome does not prevent destabilization of the G-domain by domain II. However, trigger factor, a bacterial nascent chain binding chaperone, binds to and sequesters domain II, protecting the folded state of the G-domain (Liu et al., 2019a). Controlled release of domain II once it is fully synthesized and capable of productive folding may then ensure correct structure acquisition.

It is unclear how general the denaturing effect of an unfolded domain on a folded neighbor is. As for other aspects of co-translational folding, computer simulations and theoretical models (reviewed elsewhere, see ref. (Trovato and O'Brien, 2016)) are poised to complement experimental observations in dissecting mechanistic details. Domains G and II from EF-G so far represent the only experimental example of destabilization by unfolded polypeptide in a natural multi-domain protein. However, destabilizing effects that are frequently observed in fusion proteins may be manifestations of this effect. Fluorescent proteins fused to other proteins like engrailed homeodomain or phosphoglycerate kinase have been observed to reduce the stability of the protein (Sokolovski et al., 2015; Dave et al., 2016). C-terminal disordered regions show destabilizing effects in several proteins. For instance, a tail present in the UGDH enzyme dimer reduces thermal stability by 3.6 °C (Keul et al., 2018). The presence of extended C-terminal tail also increases conformational flexibility and destabilizes the UVR8 plant photoreceptor protein (Camacho et al., 2019). Destabilizing effects are also well-known for signal peptides of secreted proteins, which effectively prevent the formation of stable tertiary structures (Randall and Hardy, 1986; Park et al., 1988; Singh et al., 2013). It thus seems possible that destabilization of folded domains by unfolded neighbors is a general phenomenon.

## Conclusion

Although multi-domain proteins make up a large proportion of proteomes, a mechanistic understanding of their unique folding pathways is still lacking, in part due to experimental challenges. Methodological advances in the past few years have fueled progress in our understanding of how large proteins fold, illustrated by the examples highlighted here. Some of the complexity that results from interactions of folded and unfolded domains during multi-domain protein biogenesis is now beginning to be unraveled. The recently developed approach of following protein folding *in vivo* using arrest peptides (Goldman et al., 2015; Marino et al., 2016; Chen et al., 2020) offers exciting opportunities for complementing biophysical *in vitro* experiments. However, despite recent advances, many questions regarding multi-domain protein folding remain: How are protein synthesis and folding tuned to each other to avoid the formation of non-functional or toxic products? How do the functional requirements for conformational flexibility compete with folding and interdomain interactions? And how do molecular chaperones rescue misfolded states of multi-domain proteins? Studies on diverse sets of authentic multi-domain proteins *in vivo* and *in vitro* will be required to define general principles of their folding.
